# Whole-genome sequencing reveals changes in genomic diversity and distinctive repertoires of T3SS and T6SS effector candidates in Chilean clinical *Campylobacter* strains

**DOI:** 10.3389/fcimb.2023.1208825

**Published:** 2023-07-13

**Authors:** Assaf Katz, Lorena Porte, Thomas Weitzel, Carmen Varela, Cristina Muñoz-Rehbein, Juan A. Ugalde, Christopher Grim, Narjol González-Escalona, Carlos J. Blondel, Verónica Bravo

**Affiliations:** ^1^ Programa de Biología Celular y Molecular, ICBM, Facultad de Medicina, Universidad de Chile, Santiago, Chile; ^2^ Laboratorio Clínico, Clínica Alemana de Santiago, Facultad de Medicina, Clínica Alemana, Universidad del Desarrollo, Santiago, Chile; ^3^ Instituto de Ciencias e Innovación en Medicina (ICIM), Facultad de Medicina Clínica Alemana, Universidad del Desarrollo, Santiago, Chile; ^4^ Center for Bioinformatics and Integrative Biology, Facultad de Ciencias de la Vida, Universidad Andrés Bello, Santiago, Chile; ^5^ Center for Food Safety and Applied Nutrition, Food and Drug Administration, College Park, MD, United States; ^6^ Instituto de Ciencias Biomédicas, Facultad de Medicina y Facultad de Ciencias de la Vida, Universidad Andrés Bello, Santiago, Chile; ^7^ Centro de Investigaciones Biomédicas y Aplicadas (CIBAP), Escuela de Medicina, Facultad de Ciencias Médicas, Universidad de Santiago de Chile, Santiago, Chile

**Keywords:** molecular epidemiology, sequence analysis, campylobacteriosis, clonal complexes, antimicrobial resistance, T6SS, fT3SS

## Abstract

*Campylobacter* is the leading cause of bacterial gastroenteritis worldwide and an emerging and neglected pathogen in South America. This zoonotic pathogen colonizes the gastrointestinal tract of a wide range of mammals and birds, with poultry as the most important reservoir for human infections. Apart from its high morbidity rates, the emergence of resistant strains is of global concern. The aims of this work were to determine genetic diversity, presence of antimicrobial resistance determinants and virulence potential of *Campylobacter* spp. isolated from patients with acute gastrointestinal disease at ‘Clinica Alemana’, Santiago de Chile. The study considered the isolation of *Campylobacter* spp., from stool samples during a 20-month period (January 2020 to September 2021). We sequenced (NextSeq, Illumina) and performed an in-depth analysis of the genome sequences of 88 *Campylobacter jejuni* and 2 *Campylobacter* coli strains isolated from clinical samples in Chile. We identified a high genetic diversity among C. je*juni* strains and the emergence of prevalent clonal complexes, which were not identified in our previous reports. While ~40% of strains harbored a mutation in the gyrA gene associated with fluoroquinolone resistance, no macrolide-resistance determinants were detected. Interestingly, gene clusters encoding virulence factors such as the T6SS or genes associated with long-term sequelae such as Guillain-Barré syndrome showed lineage-relatedness. In addition, our analysis revealed a high degree of variability regarding the presence of fT3SS and T6SS effector proteins in comparison to type strains 81-176, F38011, and NCTC 11168 and 488. Our study provides important insights into the molecular epidemiology of this emerging foodborne pathogen. In addition, the differences observed regarding the repertoire of fT3SS and T6SS effector proteins could have an impact on the pathogenic potential and transmissibility of these Latin American isolates, posing another challenge in characterizing the infection dynamics of this emergent and neglected bacterial pathogen.

## 1 Introduction

*Campylobacter* spp. is one of the most frequent causes of human bacterial gastrointestinal infections worldwide (Acheson and Allos, 2001; Kaakoush et al., 2015). Of the 31 species, which so far have been described (Costa and Iraola, 2019), *Campylobacter jejuni* and *Campylobacter coli* are the most prevalent species isolated from humans (Butzler, 2004). Infections are mainly associated with the consumption of contaminated raw or undercooked poultry (Humphrey et al., 2007). Campylobacteriosis typically manifests as self-limiting diarrhea; however, in some cases, it might cause long-term sequelae such as Miller Fisher and Guillain-Barré syndrome (GBS) (Finsterer, 2022). Antimicrobial therapy, recommended for prolonged or severe manifestations, is hampered by increasing resistance to different groups of antimicrobials in isolates from human and animal sources worldwide (Rivera et al., 2011; Tang et al., 2017; World Health Organization E, 2017).

In contrast to other gastrointestinal pathogens, *Campylobacter* does not rely solely on classical virulence factors such as toxins and adhesins (Elmi et al., 2021). Virulence is mainly based on other phenotypical characteristics such as motility, metabolic potential, and the modification of surface structures (capsule and lipooligosaccharides (LOS)) (Kreling et al., 2020). In addition, recent studies on *C. jejuni* virulence highlighted the importance of protein secretion systems such as the flagellar Type III Secretion System (fT3SS) and the Type VI Secretion System (T6SS) to *C. jejuni* virulence (Lertpiriyapong et al., 2012; Bleumink-Pluym et al., 2013; Agnetti et al., 2019; Gabbert et al., 2023). Flagellar Type III Secretion System (fT3SS) comprises an MS ring and C ring surrounding the system core in the inner membrane and a rod and hook structure that crosses the periplasm and connects to the flagellar filament extended towards the exterior of the bacterial cell (Burnham and Hendrixson, 2018). *C. jejuni* translocates at least ten effector proteins, contributing *in vitro* to host-cell adhesion and invasion and *in vivo* to colonization and immune modulation of infected chickens (Konkel et al., 1999; Barrero-Tobon and Hendrixson, 2012). Notably, a recent bioinformatic analysis predicted 57 putative fT3SS effector candidates encoded in the type strains F38011, NCTC 11168, and 81-176 (Gabbert et al., 2023), suggesting that the total number of effectors translocated by *C. jejuni* is more extensive than previously estimated.

Some *C. jejuni* strains also encode for a T6SS (Robinson et al., 2021) which is a multiprotein contractile injection system that delivers effector proteins into target cells (Ho et al., 2014; Zoued et al., 2014; Coulthurst, 2019). While some T6SS effectors target only bacteria, others target bacterial and eukaryotic cells (Durand et al., 2014; Alcoforado Diniz et al., 2015; Hachani et al., 2016). By employing the T6SS against bacterial rivals, several strains have been demonstrated competitive advantages in various environments, such as the plant surface or the intestinal epithelium (Bernal et al., 2018; Wu et al., 2018; Allsopp et al., 2020). Other enteric pathogens such as *Salmonella, Shigella*, and *Vibrio* use the T6SS to colonize the gut (Sana et al., 2017; Chassaing and Cascales, 2018), while some of the Bacteroides strains of the gut microbiota use their T6SSs to antagonize certain pathogenic species (Coyne and Comstock, 2019). In *C. jejuni*, the T6SS contribute to a variety of *in vitro* and *in vivo* phenotypes, ranging from host cell adherence, cellular invasion, resistance to bile salts or oxidative stress, and interbacterial competition to the ability to colonize both mouse and chicken *in vivo* infection models efficiently (Lertpiriyapong et al., 2012; Bleumink-Pluym et al., 2013; Liaw et al., 2019). No T6SS effector protein has been fully characterized for *Campylobacter*, but recent bioinformatic analyzes of the genome of *C. jejuni* strain 488 have predicted the presence of at least 15 effector candidates (Robinson et al., 2021; Robinson et al., 2022).

Despite the importance of *Campylobacter* as an emerging enteric pathogen, its prevalence in low and middle-income countries is widely unknown (Platts-Mills and Kosek, 2014; Kaakoush et al., 2015; Collado et al., 2018; Levican et al., 2019; Collado, 2020). Recent reports in the Latin American region have shown a high prevalence of human campylobacteriosis (Porte et al., 2023), including significant outbreaks causing long-term autoimmune sequelae such as GBS (Ramos et al., 2021; Quino et al., 2022). The absence of widely globally distributed lineages in some areas of Latin American countries such as Peru (Pascoe et al., 2020) emphasizes the need to gain a deeper insight into the molecular epidemiology and characteristics of the predominance of particular genotypes in the region. In addition, the variability of virulence and pathogenic potential of Latin American isolates is vastly unknown, which affects our understanding of the dynamics of this neglected bacterial pathogen.

In this context and continuing with our prior efforts to understand the genomic epidemiology of *Campylobacter* spp in Chile, this work provides a genetic characterization of clinical *Campylobacter* strains from patients with gastrointestinal disease in Santiago. In addition to the most prevalent lineages, we described Clonal Complexes (CCs) not previously identified in the city as well as virulence factors over-represented in some lineages. In addition, our analysis revealed that Chilean clinical strains harbor distinct combinations of T3SS and T6SS effector protein candidates, previously identified in *C. jejuni* reference strains 81-176, F38011, NCTC 11168 and 488.

Our work increases our understanding of the genomic epidemiology of this emerging pathogen in Chile. It also highlights the variability of the repertoire of T3SS and T6SS effector proteins encoded in these clinical strains, which may have important implications for the environmental fitness, transmission, and pathogenic potential of these strains.

## 2 Materials and methods

### 2.1 Bacterial cultures and DNA extraction

*Campylobacter* strains derived from clinical stool samples obtained from patients with gastrointestinal disease, were tested for bacterial pathogens at ‘Clínica Alemana’, a private non-for-profit healthcare center in Santiago, Chile, between January 2020 and September 2021 (Porte et al., 2016). Briefly, isolates were initially cultivated on CASA agar (BioMerieux®) and identified by MALDI-TOF mass spectrometry (VITEK MS, BioMerieux®). Then, each isolate was stored at -80°C in cryotubes (Cryobank, Mast Group Ltd.). For DNA extraction, strains were thawed and cultivated overnight on Mueller-Hinton 5% sheep blood agar plates at 42°C under microaerophilic conditions (Biomerieux®). Genomic DNA was extracted using the DNeasy blood and tissue kit (Qiagen®) following the manufacturer´s protocol.

### 2.2 Genome data set, sequencing, and annotation

From a total of 116 strains isolated in the period mentioned above, we randomly chose 90 *Campylobacter* spp isolates for sequencing. The genome sequence dataset analyzed in this study is listed in **Supplementary Table S1**. Sequencing libraries were prepared with the Nextera DNA Prep library kit and DNA/RNA UD sequencing indexes (Illumina, San Diego, CA USA), with 100-200 ng of input DNA. Sequencing libraries were prepared on a Sciclone G3 NGSx iQ liquid handling workstation (Perkin Elmer, Waltham, MA USA). Genomes were sequenced on a NextSeq 2000 (Illumina, San Diego, CA USA), using a P2 300 cycle (2 × 150-bp paired-end) chemistry sequencing, according to the manufacturer’s instructions, aiming for a genome coverage of 60x-600x. Default parameters were used for all software unless otherwise specified. The Illumina paired reads were analyzed with the CLC Genomics Workbench v9.5.2 (Qiagen), assessed for quality (Q>30) with the quality control tool, and trimmed (adapter trimming, quality trimming, and length trimming) with the trim sequences tool. The trimmed data was *de novo* assembled using CLC Genomics Workbench and a minimum contig size threshold of 500 bp.

A subset of strains was chosen for long-read sequencing (**Supplementary Table S1**). The selected 7 strains were: CFSAN122719 (SAMN31838813), CFSAN122739 (SAMN31838830), CFSAN122748 (SAMN31838839), CFSAN122778 (SAMN31838866), CFSAN122786 (SAMN31838874), CFSAN122787 (SAMN31838875), and CFSAN122822 (SAMN31838909). These strains were selected according to their virulence factors distribution pattern. Thus, strains CFSAN122719 and CFSAN122786 were closed to confirm the presence of pVir plasmid and strains CFSAN122739, CFSAN122748, CFSAN122778 and CFSAN122787 to determine the presence/absence of genes encoding structural and effectors proteins of the T6SS. Those isolates belong to different STs. Finally, strain CFSAN122822 was chosen because it belongs to the specie *C. coli* which has been poorly characterized in the country.

Long reads were generated on a GridION sequencer (Nanopore Technologies, Oxford, UK). Sequencing libraries were prepared using the rapid barcoding sequencing kit (SQK-RBK004). Seven libraries were pooled and run in a FLO-MIN106 (R9.4.1) flow cell for 48 hours, according to the manufacturer’s instructions. Base-calling was performed using default settings (MinKNOW v22.10.5, Guppy v6.3.8). Sequencing outputs was 2.50 Gb (quality score, 11.29; N50, 3,405 bp; total reads, 150.727), for an estimated average genome coverage of 30 to 50×. Reads of <2,000 bp and a quality score of <7 were discarded for downstream analysis. The short-read whole-genome sequence for these strains were generated as described earlier using a NextSeq sequencer. The final complete genome sequences (comprising the chromosome and plasmid, when present) were obtained using a previously described pipeline (Maguire et al., 2021). Unicycler software v0.4.8 (Wick et al., 2017) was utilized to perform hybrid assembly and short and long-read sequencing output and resulted in completely or nearly completely closed and circular genomes.

The genomes were annotated using Prokka (Seemann, 2014) with the genomes of *C. coli* aerotolerant strain OR12 (accID GCF_002024185.1) and *C. jejuni strain* NCTC 11168 (accID GCF_000009085.1) as references and by forcing GenBank compliance.

### 2.3 MLST and cgMLST analysis

For MLST and cgMLST, sequencing reads were mapped against an MLST scheme based on seven housekeeping genes and 637 core and 958 accessory gene cgMLST scheme (Cody et al., 2017), using Ridom SeqSphere software (Münster, Germany). A phylogenetic comparison of the 90 *Campylobacter* genome sequences was performed using a neighbor-joining (NJ) tree based on a distance matrix of the core genomes of all the strains. STs and clonal complexes (CCs) were determined after automated allele submission to the PubMLST server for *Campylobacter* MLST (https://pubmlst.org/campylobacter/) (Jolley et al., 2018). Further annotations and visualizations of trees were performed using iTOL v.6 (Letunic and Bork, 2019).

### 2.4 Virulome and resistome analysis

The bacterial virulome was determined by genome hits against the genes of the virulence factor database (VFDB) (Liu et al., 2019) using the minimum length coverage of 60% aligned to the reference and 85% of identity. Antimicrobial resistance determinants were screened in each *Campylobacter* genome using ABRicate (Seemann, 2020), which includes the Resfinder (Florensa et al., 2022), CARD (Alcock et al., 2022), ARG-ANNOT (Gupta et al., 2014) and NCBI ARRGD (Feldgarden et al., 2019) databases. *gyrA* and 23S RNA genes point mutations were determined using Resfinder (Florensa et al., 2022). BLAST analysis was performed as previously described (Bravo et al., 2021) and genetic clusters were visualized using EasyFig (Sullivan et al., 2011). In both virulome and resistome analysis, short-reads assemblies of each strain were used. To predict the source of the *Campylobacter* strains, first cgMLST profiles were assigned using chewBBACA (Silva et al., 2018) using the *Campylobacter* pubMLST database (Cody et al., 2017). The resulting cgMLST profile was processed using aiSource (Arning et al., 2021) to predict source attribution of the isolate. For the analysis of the distribution of T3SS and T6SS effector candidates, the nucleotidic sequences of genes coding for each previously described effector protein (Gabbert et al., 2023) were used as baits in BLAST analysis, as described above. Hierarchical clustering analysis was performed using the versatile matrix visualization and analysis software Morpheus using a presence/absence matrix of each effector candidate (**Table S2**). The hierarchical clustering recursively merges objects based on their pair-wise distance and this results in dendrogram where profiles which are more similar will cluster together in closer branches (one minus Pearson correlation, average linkage method) (https://software.broadinstitute.org/morpheus).

### 2.5 Comparative genomic and sequence analysis

Genome nucleotide sequences were visualized with the Artemis 18.1 software (Carver et al., 2012) and analyzed with the Artemis Comparison Tool (ACT) release 6 (Carver et al., 2005), the multiple aligner Mauve (Darling et al., 2010) and EasyFig v2.2.2 software (Sullivan et al., 2011). Putative effectors were analyzed with the structure-based homology tool HHpred (Zimmermann et al., 2018). For the pangenome analysis of pVir plasmids, we performed long-read sequencing of CFSAN122719 and CFSAN122748 strains, following the protocol described above. Nucleotide plasmid sequences were compared to the pVir plasmid nucleotide sequence of *C. jejuni* 81-176. For that comparison we used Roary (Page et al., 2015), setting a minimum percentage identity of 90% for BLASTp.

For the identification of novel T6SS effector candidates, the amino acid sequence of each ORF was analyzed with the Bastion6 pipeline (Wang et al., 2018), the DeepTMHTMM software (Hallgren et al., 2022) to predict putative transmembrane domains, the structure-based homology tool HHpred (Zimmermann et al., 2018) and the PROSITE, NCBI-CDD, Motif-finder, and Pfam databases to identify putative conserved functional domains (Kanehisa, 2002; Sigrist et al., 2012; Finn et al., 2014; Lu et al., 2020). For this analysis, long-read assemblies were used.

## 3 Results

### 3.1 cgMLST analysis and phylogenetic distribution of Chilean *C. jejuni* strains

**Figure 1:**
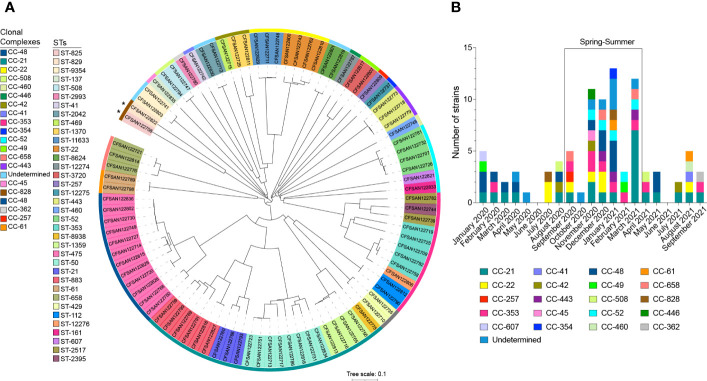
Phylogenetic Analysis and distribution of CC over time. **(A)** cgMLST phylogenetic analysis of 90 clinical *Campylobacter* strains isolated from patients with gastrointestinal disease in Santiago, Chile. Neighbor joining (NJ) tree was constructed with RIDOM SeqSphere^+^ using *Campylobacter jejuni* subsp. *jejuni* NCTC 11168 as reference genome. The outer colored strip represents the different clonal complexes described in the study. The colored background of each strain name denotes STs. iTOL (Letunic and Bork, 2019) was used for tree visualization. Asterisks denotes *C coli* isolates. **(B)** Monthly distribution of CCs in clinical *Campylobacter* strains collected in a 20 months period (January 2020 to September 2021) in Santiago, Chile. Colored bars represent each clonal complex described in the study.

**Figure 2:**
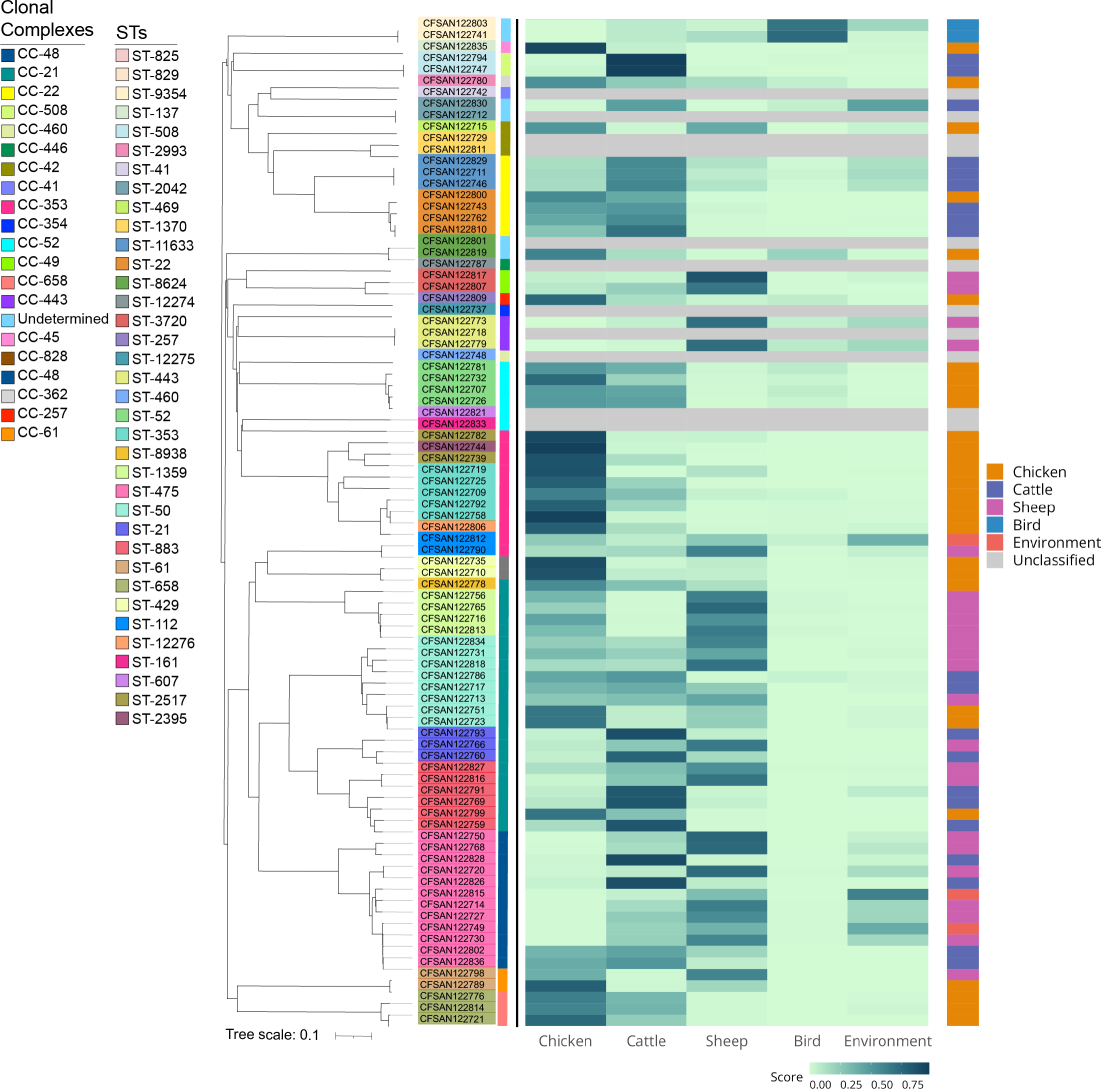
Source attribution of clinical *Campylobacter* isolates. The source of each isolate was predicted based on cgMLST profiles processed using aiSource (Arning et al., 2021). The figure shows a heat map based on the probability of attribution to the tested sources for each strain ordered based on the NJ tree. The colored bar on the right indicates the predicted source while the bar in the left denotes the CC for each strain.

The study included a total of 90 clinical *Campylobacter* strains of which 88 (97.8%) were *C. jejuni* and 2 (2.2%) were *C. coli*. The cgMLST analysis showed a high degree of diversity within the 90 strains analyzed. These strains belong to 49 sequence types (STs), identified within 22 clonal complexes (CCs) (**Figure 1A**). More than 50% of isolates (n=53) belonged to the worldwide prevalent lineages CC-353, CC-21, CC-22, and CC-48 (**Figure 1A**; **Supplementary Table S3**). ST-475 belonging to CC-48, was the most frequent STs identified in this study (n=12, 13.3%). Based on previous reports we cataloged all CCs based on the most probable isolation source. CC-21 was the most abundant and diverse among host-generalist clonal complexes (Dearlove et al., 2016) accounting for 22 isolates of 5 different STs with a predominance of ST-883 (6 isolates) and ST-50 (8 isolates) (**Figure 1A**; **Supplementary Table S3**). CC-353, a chicken-associated complex (Sheppard et al., 2011), was the most prevalent host-specialist CC (representing 11.1% of the isolates; n=10), followed by the cattle-associated CC-22 (Epping et al., 2021) (7.8%; n=7). In agreement with these observations, a source attribution analysis confirmed that the most prevalent CCs found in this study (CC-21, CC-353- CC-22) are related to known animal sources (**Figure 2**).

Several studies have reported that campylobacteriosis cases follow a distinctive seasonality, with peak cases during the spring and summer (Nylen et al., 2002). Most strains (62.2%; n=56) were isolated during those seasons (September to March in Chile) (**Figure 1B**). The most prevalent strains followed a similar trend. While strains of CC-353 and CC-48 were mostly isolated during the spring-summer period, CC-21 was present over the whole period of study with a peak in summer (**Figure 1B**).

### 3.2 Resistome analysis of Chilean *C. jejuni* strains clinical strains

**Figure 3:**
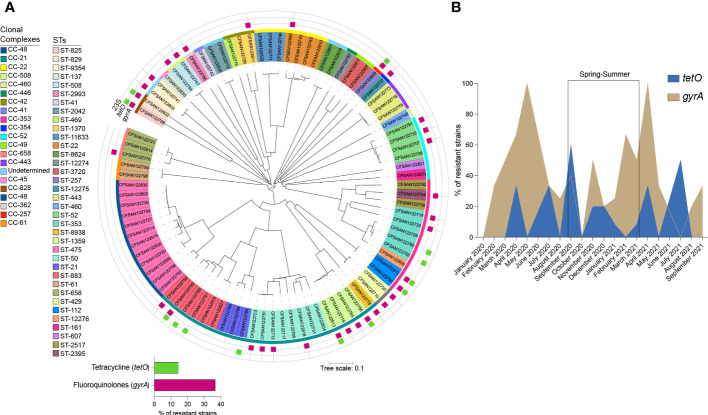
Presence of genetic resistance determinants amongst clinical *Campylobacter* strains collected in Santiago, Chile. **(A)** NJ tree indicating the presence of resistance determinants among *Campylobacter* spp isolates. Resistance determinants are denoted by colored squares surrounding the tree and grouped according to the spectrum of action: Fluoroquinolones (magenta), macrolides, not detected (light blue), and tetracycline (pea). **(B)** Monthly distribution of fluoroquinolones (khaki) and tetracycline (royal blue) determinants.

Antimicrobial determinants in the genome sequences of the 90 *Campylobacter* strains were identified using Abricate software and Resfinder (Camacho et al., 2009; Zankari et al., 2017; Bortolaia et al., 2020; Seemann, 2020). None of the analyzed isolates encoded for the mutations A2058C and A2059G in the 23S RNA gene, which are associated with macrolide resistance (Tang et al., 2017) (**Figure 3A**). The C257T point mutation in the quinolone resistance-determining regions (QRDR) of the *gyrA* gene, which confers class-wide resistance to fluoroquinolones, was detected in 36.7% of isolates (n=33) (Porte et al., 2023). As shown in **Figure 3A**, all ST-1359 strains harbored this mutation, while all the isolates from the most common ST (ST-475) are predicted to be sensitive to this group of antimicrobials. The analysis of genomes grouped by month of isolation, revealed an increase in the isolates predicted to be resistant to fluoroquinolones in April/May, both for strains isolated in 2020 and 2021 (**Figure 3B**).

The *tetO* gene, which encodes for the ribosomal protection protein involved in tetracycline resistance, was present in 14.4% of the analyzed genomes (n=13). ß-lactamase encoding genes (*blaOXA-61*, *blaOXA-193*, *blaOXA-450*, and *blaOXA-605*), were also prevalent among the *Campylobacter* genomes (**Supplementary Table S4**). The analysis of the genome sequence of a predicted multi-resistant strain (strain CFSAN122769), revealed the presence of a DNA fragment that encodes genes conferring resistance to aminoglycosides (*aph(2″)-If*, *aac(6’)-Im, ant (6*)*-Ia*), and tetracycline (*tetO*). Additionally, multidrug efflux pump genes *cmeABC* were detected in all the *C. jejuni* isolates.

### 3.3 Analysis of the distribution of virulence-related genes in Chilean *C. jejuni* strains

**Figure 4:**
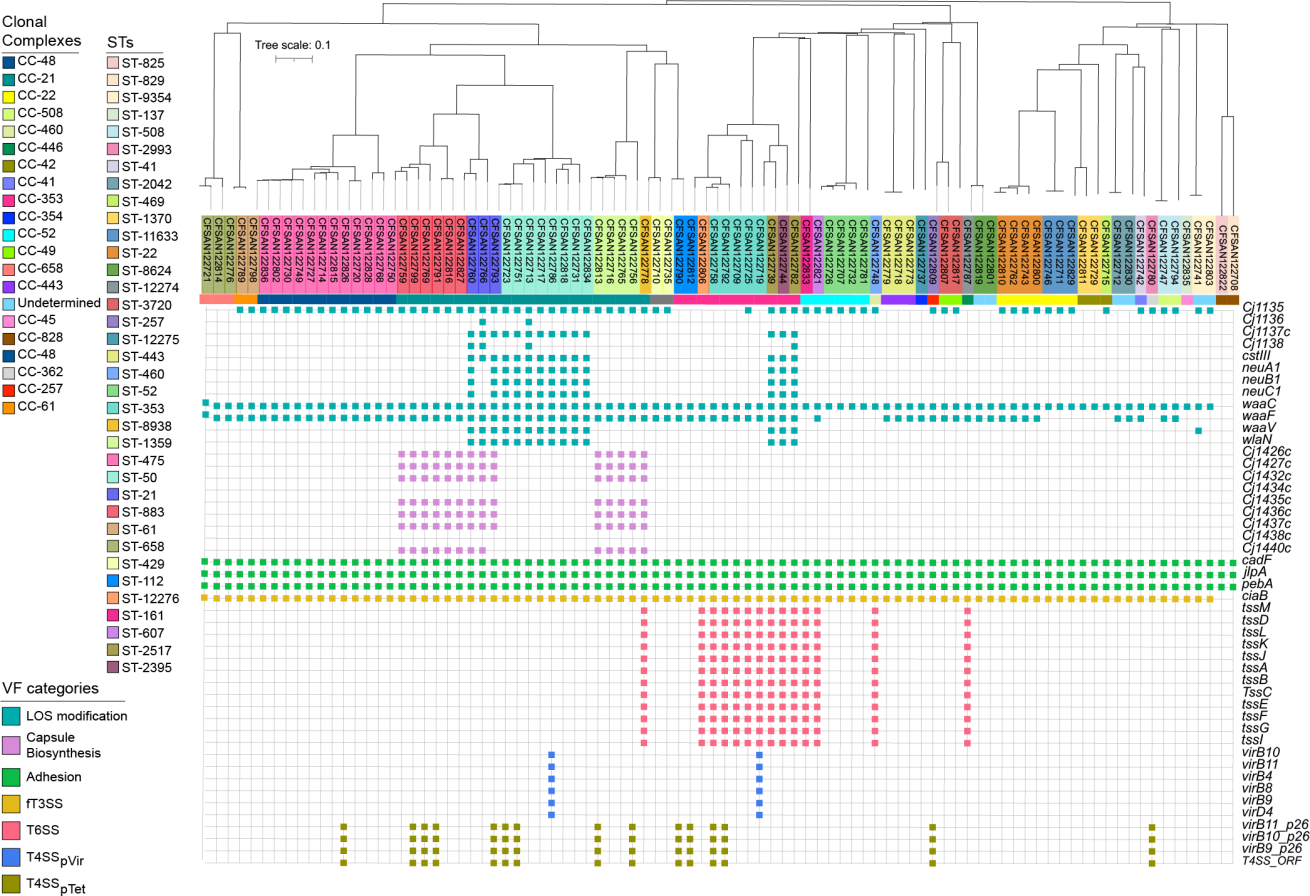
Virulome analysis of clinical *Campylobacter* strains isolated from patients with gastroenteritis. The colored strip represents the CCs identified in this group of strains. Virulence determinants were divided into seven different categories and highlighted by colored squares: LOS modification (teal), capsule modification (lavender), adhesion (spring green), invasion (golden), T6SS (pale red), T4SS_pVir_ (azure) and T4SS_pTet_ (olive).

The distribution of virulence determinants is shown in **Figure 4**; **Supplementary Table S5**. Genes were classified according to their associated functions, including capsule synthesis, toxins production, LOS synthesis, fT3SS, T4SS, and T6SS.

Adhesion associated genes (*cadF*, *jlpA*, *pebA*) as well as the cytotoxin-encoding cluster *cdtABC* were detected in all the analyzed genome sequences (**Figure 4**). Genes involved in capsule biosynthesis (*Cj1426c*, *Cj1427c*, *Cj1432c*, *Cj1434c*, *Cj1435c*, *Cj1436c*, *Cj1437c*, *Cj1438c*, *Cj1440c*) were only present in strains belonging to CC-21, particularly in all isolates from ST-883, ST-21, and ST-1359. Additionally, *cstIII*, *neuABC* and *wlaN*, which have been involved in molecular mimicry (Linton et al., 2000; Linton et al., 2002; Culebro et al., 2018), were detected in 100% of the strains belonging to ST-50 and ST-21.

**Figure 5:**
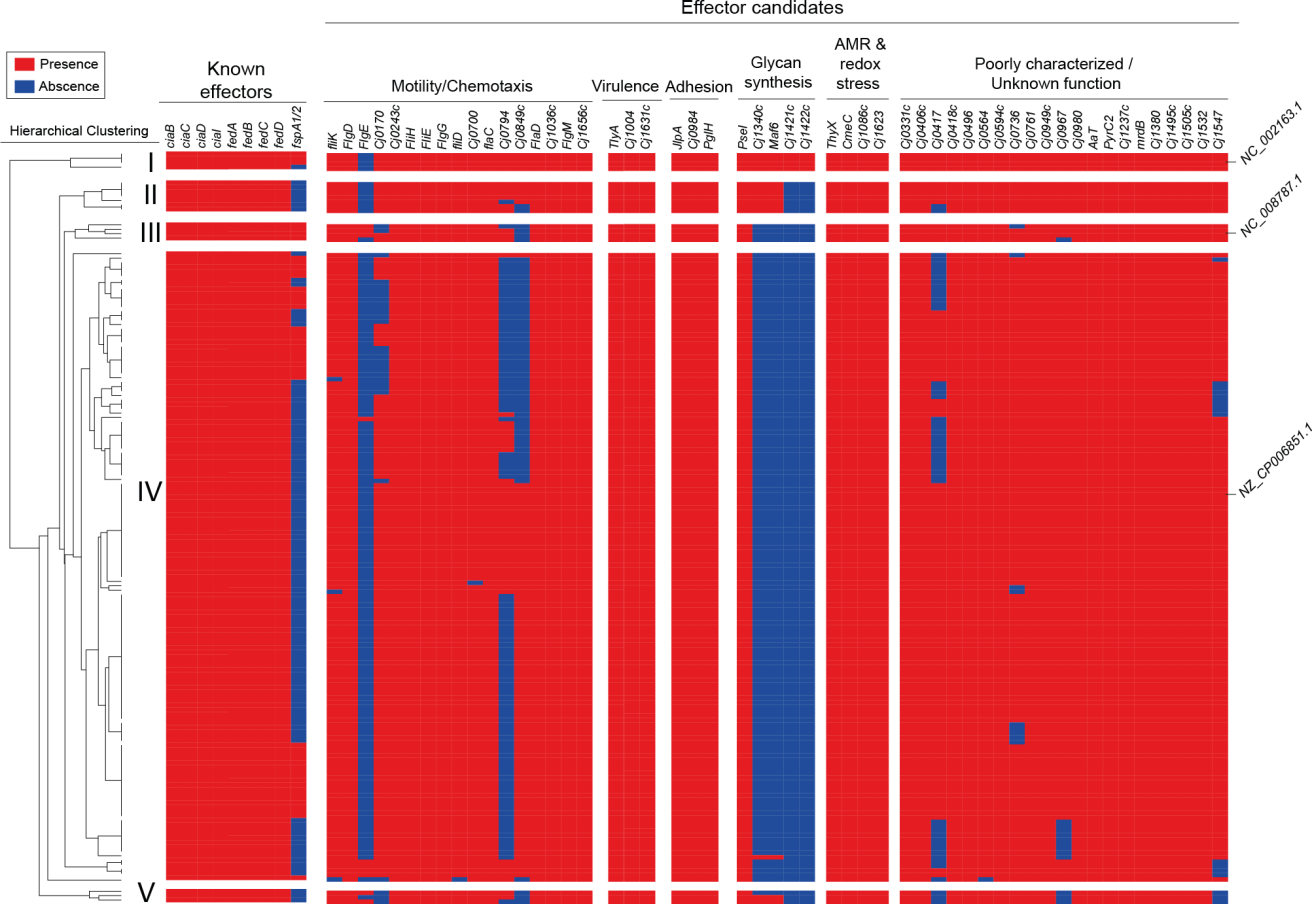
Distribution and hierarchical clustering analysis of T3SS components in clinical *C. jejuni* strains. Clustering analysis was performed using the versatile matrix visualization and analysis software Morpheus (one minus Pearson correlation, average linkage method). The red and blue boxes represent the presence and absence of individual genes, respectively.

Regarding the fT3SS, in each of the strains we detected the genes encoding the major structural components such as the flagellar basal body (*flgB* and *flgC*), hook (*flgE2*), filament (*flaA* and *flaB*), as well as genes encoding essential components for flagellar protein export (*flhB* and *fliI*) (**Supplementary Table S5**). This suggests that each of the strains encode a functional fT3SS. To determine the presence and distribution of the known and putative fT3SS effectors among Chilean strains, we generated a local database with the genome assemblies of genome sequences of *C. jejuni* from our previous (Bravo et al., 2020b; Bravo et al., 2020a) and current work (total of 171 strains). Our analysis also included the *C. jejuni* type strains F38011, NCTC 11168, and 81-176. As shown in **Figure 5**, there is a differential distribution of an important group of effector proteins in the Chilean isolates. Hierarchical clustering analysis revealed five subgroups with distinct repertoires of effector protein candidates (**Figure 5**). Interestingly, most Chilean clinical strains cluster with type strain F38011, while only a few clustered with type strains NCTC 11168 and 81-176. In addition, two subgroups (II and V) did not cluster with any type strain.

Every Chilean isolate harbored previously described effectors, except for FspA1 and FspA2 that were absent in several isolates (**Figure 5**). The largest diversity was observed in terms of the presence of the effector candidates identified by Gabbert et al. (Gabbert et al., 2023), especially in the motility/chemotaxis category. Predicted effectors related to glycan synthesis were absent in most Chilean isolates (**Figure 5**). These results highlight the diversity regarding fT3SS effectors in these Chilean strains.

Another protein secretion system linked to *C. jejuni* virulence is the T4SS. This system is a membrane-associated complex used by bacteria to deliver molecules to several target cells in a contact-dependent manner and is usually encoded in the pTet family plasmids of *Campylobacter* (Bacon et al., 2000; Marasini et al., 2018). In the current study, this system was associated with several STs, most of them belonging to CC-21 and CC-353 (**Figure 4**). However, not all isolates of these STs encoded this secretion system. Additionally, we identified two strains harboring the pVir plasmid, associated with increased invasion of epithelial cells in *C. jejuni* 81-176 strain (Cody et al., 2012). While the presence of this plasmid is low among clinical isolates worldwide (Panzenhagen et al., 2021), this is the first report of its presence in clinical strains from Chile. Sequence-based analysis showed that these plasmids were more similar to pVir isolated from Japan (strain THJ065) (**Supplementary Figure S1**). The main differences between pVir isolated from Chilean strains and strain 81-176 is a set of hypothetical proteins and the DinJ-YafQ Type II toxin-antitoxin (TA) module in the Chilean strains (**Supplementary Table S6**).

As mentioned above, the T6SS has emerged as a novel fitness and virulence factor in *C. jejuni*, contributing to host colonization and resistance to oxidative stress (Liaw et al., 2019). Our bioinformatic analysis identified putative T6SS gene clusters (based on the detection of each of the major T6SS structural components) in 14 C*. jejuni* strains (~16%), more than 2-fold increase compared to our previous study from the same health center (Bravo et al., 2021) (**Supplementary Table S5**). Interestingly, 64% (n=9) of the T6SS positive strains of this study belonged to the CC-353 clonal complex, which is generally attributed to a chicken source.

**Figure 6:**
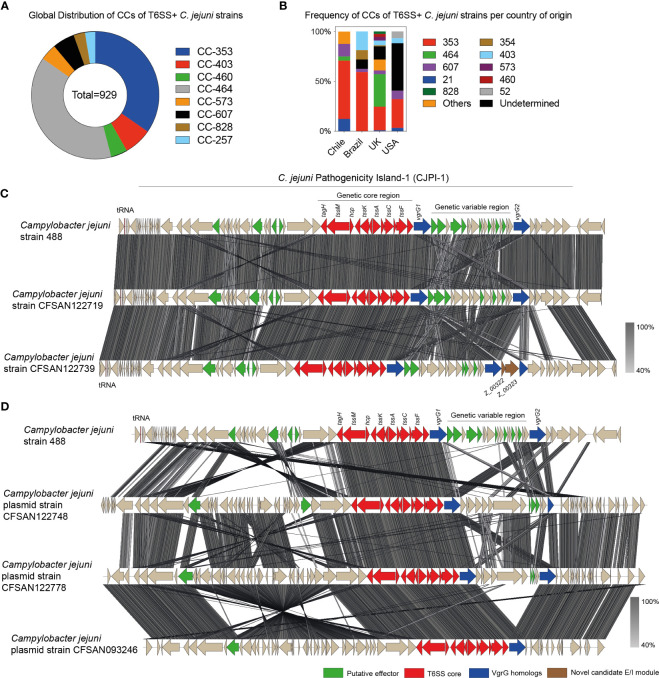
Clonal complex distribution and genomic comparative analysis of *C jejuni* encoding T6SSs. **(A)** Analysis of the global distribution of CC of T6SS positive *C jejuni* strains. **(B)** Frequency analysis of the CCs of T6SS positive *C jejuni* strains per country of origin. **(C)** and **(D)** Comparison of the T6SS gene cluster in *C jejuni* 488 with the T6SS gene clusters encoded in either the chromosome C or plasmids **(D)** from clinical strains isolated from Chile. tBLASTx alignments were performed and visualized using Easyfig. Genes encoding different T6SS-related components are highlighted in color.

To determine and compare this distribution with other countries, we analyzed the databases of van Vliet et al. and Robinson et al. and included the CC and ST of every *C. jejuni* strain from Chile sequenced to date (van Vliet et al., 2021; Robinson et al., 2022). The analysis corroborated the global representation of CC-464, CC-353, CC-573, and CC-403 (**Figure 6A**). However, an analysis based on the country of origin revealed important differences between Latin American countries such as Chile and Brazil and countries from the Northern Hemisphere, such as the UK and the United States. While in the UK the two most frequent T6SS positive CCs are CC-464 (32.6%) and CC-353 (23.6%), in Chile and Brazil, only the CC-353 is found at a high frequency (58.3% and 59.4%, respectively), with very few strains from CC-464 found only in Chile (4.2%). CC-464 is also not frequent in strains from the United States and CC-353 is present only in 28,8% of strains (**Figure 6B**).

To gain insight into the genomic context of the putative T6SS gene clusters, we performed a comparative genomic analysis between the T6SS gene cluster of *C. jejuni* strain 488 and the T6SS gene clusters of 4 C*. jejuni* strains with closed or near-finished genomes (genomes with contigs large enough to contain complete T6SS gene clusters) (strains CFSAN122719, CFSAN122739, CFSAN122748 and CFSAN122778). As shown in **Figure 6C** there was a high degree of synteny between the chromosomally encoded T6SS clusters. This synteny was also observed in the variable region described initially in strain 488 and located between two copies of the *vgrG* gene (**Figure 6C**). Strain CFSAN122739 harbored an additional copy of *vgrG* and several additional ORFs flanking these gene encoding diverse hypothetical proteins. Our analysis showed that the T6SS gene clusters of strains CFSAN122719 and CFSAN122739 are located in the chromosome within the CjiE3 conjugative element (just as in the 488 strain). Interestingly, the T6SS gene clusters of strains CFSAN122748 and CFSAN122778 were encoded in distinct plasmids (**Figure 6D**) and were similar to the plasmid of strain CFSAN093246 previously identified in the same clinic (Bravo et al., 2021), although with difference in the genetic variable region of the T6SS gene cluster. A recent bioinformatic survey identified that *Campylobacter* T6SS gene clusters could be classified into three distinct cluster organizations (types I, II, and III). Analysis of T6SS genetic organization revealed that each of the T6SS gene clusters identified belonged to the T6SS group I-a, the same T6SS group as the T6SS of the Brazilian type strain 488 (Robinson et al., 2021; Robinson et al., 2022). In addition, we analyzed each ORF of the chromosomally encoded T6SS gene clusters in strains CFSAN122719 and CFSAN122739 and found a novel putative effector/immunity module (ORFs Z_00322 and Z_00323) in the variable region of the T6SS gene cluster of strain CFSAN122739 (**Figure 6C**). ORF Z_00322 encodes a putative membrane protein with a transmembrane domain. It is predicted to be co-transcribed with ORF Z_00323 that possesses low homology to N-acetylmuramoyl-L-alanine amidases (**Supplementary Figure S2**), suggesting that it could target the peptidoglycan of target cells contributing to the antibacterial activity of this T6SS.

**Figure 7:**
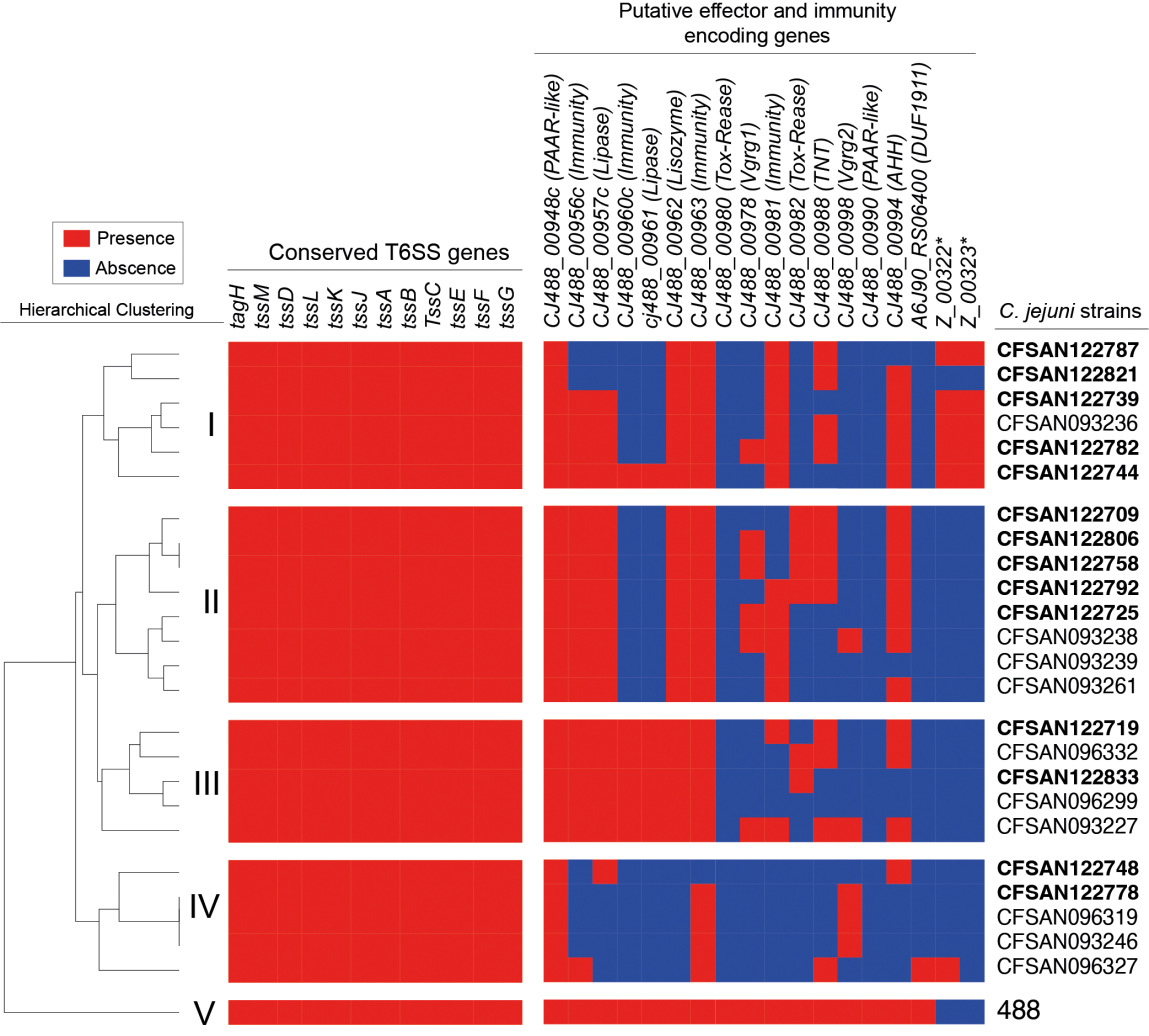
Distribution and hierarchical clustering analysis of T6SS components in *C. jejuni* strains. Clustering analysis was performed using the versatile matrix visualization and analysis software Morpheus (one minus Pearson correlation, average linkage method). The red and blue boxes represent the presence and absence of individual genes, respectively. Asterisks highlight the novel E/I module identified in this work.

To further characterize the T6SS of the Chilean clinical strains, we performed a bioinformatic screen for the presence and absence of previously described putative effectors and immunity proteins in each genome from our Chilean database (Robinson et al., 2021; Robinson et al., 2022). As shown in **Figure 7**, the hierarchical clustering analysis revealed the presence of 5 major subgroups (I to V) with different profiles of T6SS effector and immunity encoding genes. The *C. jejuni* strains from the present study clustered in the different subgroups, mostly in subgroup 1. Notably, the *C. jejuni* strain 488, in which most of the bioinformatic analysis of the T6SS have been performed, clustered in a different group (V), highlighting that the Chilean clinical strains harbor different repertoires of T6SS effector proteins in comparison to the type 488 strain. The most conserved effector candidates between strain 488 and the Chilean clinical strains where the CJ488_00948c (PAAR-like), CJ488_00957c (Lipase) and CJ488_00962 (Lisozyme) while the least conserved effectors where CJ488_00980 (Tox-Rease), CJ488_00982 (Tox-Rease) and CJ488_00990 (PAAR-like). The differential distribution of putative T6SS effector candidates suggest that these clinical strains might differ in their ability to interact with host-cells and/or compete with the host microbiota.

## 4 Discussion

### 4.1 ST-475 is the major sequence type among Chilean *C. jejuni* strains

The presented Chilean data is consistent with international studies, reporting a predominance of *C. jejuni* as the most prevalent species causing human campylobacteriosis (Acheson and Allos, 2001; World Health Organization, Food and Agriculture Organization of the United Nations, World Organisation for Animal Health, 2013; Kaakoush et al., 2015; EFSA, 2019). However, we found an important decline of *C. coli* within our study site from >10% during 2017-2019 (Bravo et al., 2021) to 2.2% in the present study. This difference might be related to the reduced mobility of the urban population of Santiago during the SARS CoV-2 pandemic, since higher prevalence of *C. coli* have been reported from rural environments (Roux et al., 2013). Remarkably, we did not identify emerging *Campylobacter* species, as reported in other South American countries such as Peru, where *C. concisus*, *C. upsaliensis*, and *Candidatus Campylobacter infans* were described recently (Garcia Bardales et al., 2022; Parker et al., 2022).

In contrast to reports from other regions of South America (Pascoe et al., 2020), the most common CCs identified in Chile have a rather global distribution (CC-21, CC-353, CC-48, CC-22) (Cobo-Díaz et al., 2021). Interestingly, we found an increased frequency and higher genetic diversity of CC-353 compared to our previous study (Bravo et al., 2021). Additionally, CC-22, which was not identified in previous studies in Santiago, emerged as a frequent lineage (**Supplementary Figure S3A**). The presence of this and other predominant CCs (CC-41, CC-48, CC-353, CC-21 among others) was previously reported in southern Chile (Collado et al., 2018). The presence of genetically related lineages in these geographically distant regions (approx. 800 km) might indicate common food-associated sources (e.g., poultry or cattle); such relatedness is supported by common phenotypic properties including antimicrobial resistance and virulence factors.

In Chile there is limited information about *Campylobacter* sources and their relationship with certain lineages However, recent genetic signatures demonstrated an association of allelic variants to host-specific lineages (Berthenet et al., 2019; Cody et al., 2019). In the current study we identified a relevant number of strains belonging to host-generalist lineages (CC-21 and CC-48), suggesting that they are capable of colonizing various animal reservoirs. Even though CC-48 has also been associated with a host-generalist lifestyle, most of the isolates within this CC belonged to the ST-475. Méric et al., 2018 performed a deep nucleotide and amino acid sequence analysis of an extensive collection of *Campylobacter* spp. isolates from multiple origins and determined an association between this ST and its high prevalence in human hosts (Méric et al., 2018). This could indicate that asymptomatic human carriers could play a role as reservoir in Chile or that there is no sufficient data about *Campylobacter* isolated from other animal sources. In fact, the source attribution analysis showed that most of the isolates from this ST are related to either environmental or ovine sources (**Figure 2**). This result is unexpected due to the low consumption of ovine-derived products in Santiago and the absence of reports associating *Campylobacter* cases to environmental transmission in the country. These data suggest the need of further epidemiological studies and active national surveillance to determine such potential reservoirs of this and other STs in Chile.

### 4.2 Resistome analysis predicts a high susceptibility to macrolides in clinical strains

This study identified 25 distinct antimicrobial resistance determinants and point mutations within the Chilean isolates. Even though a low percentage of strains are predicted to be resistant to aminoglycosides, we found diverse genetic determinants associated with this resistance, which agrees with worldwide observations (Cobo-Díaz et al., 2021). The high percentage of isolates harboring the C257T point mutation in the QRDR of *gyrA* confirmed the global trends regarding fluoroquinolones resistance in *Campylobacter* spp (World Health Organization E, 2017; Gomes et al., 2020). However, the presence of the *gyrA* mutation decreased compared to the findings of our previous work (Bravo et al., 2021). A similar trend was observed for the mutations and genetic markers related to macrolides resistance, which is the group of choice for treatment of human campylobacteriosis (Tang et al., 2017) (**Supplementary Figure S3B**). Although we lack information regarding antimicrobials used in animal production, these trends might result from a more restricted veterinary usage of these drugs in the last years.

### 4.3 Lineage-relatedness of LOS and capsular modifications genes

The virulome analysis revealed that all isolates harbored genes related to adherence and invasion (*cadF*, *jlpA*, *pebA*, *ciaB*), which is in accordance with previous reports (Redondo et al., 2019; Truccollo et al., 2021). Interestingly, virulence factors associated with lipooligosaccharide (LOS) and capsular modifications were lineage-related, i.e., *wlaN*, *neuA1*, *neuB1*, *neuBC1*, *cj1136*, and *cstIII* were only identified in strains belonging to CC-21 and CC-353, particularly ST-50, ST-21, ST-2395, and ST-2517. Guirado et al. had previously reported similar findings in *C. jejuni* strains isolated from different sources (Guirado et al., 2020), showing a high prevalence of *wlaN* and *cgtB* (related to GBS (Guirado et al., 2020)) (both involved in LOS sialylation) in CC-21 strains. The fact that these genetic determinants were found in high-prevalent lineages reinforces the importance of epidemiological surveillance in order to better respond to outbreaks.

### 4.4 First report of the pVir plasmid *C. jejuni* strains from Chile

Our study firstly reports the presence of the pVir plasmid in clinical strains from Chile having a high similarity to pVir plasmids from Japan strains. Although this plasmid is infrequent in clinical isolates worldwide and its role in pathogenesis is unclear, its presence has been associated to the occurrence of bloody stools in patients with campylobacteriosis (Tracz et al., 2005). Given its possible role in the clinical outcome, it is important to follow-up the dynamics of the bacterial population carrying this plasmid in Chile and South America.

### 4.5 Chilean clinical strains harbor different repertories of fT3SS and T6SS effector protein candidates

In addition to its role in bacterial motility, the fT3SS is one of the major virulence factors of *C. jejuni* (Gabbert et al., 2023). Our analysis showed the presence of the fT3SS in every strain analyzed (**Supplementary Table S5**), highlighting its importance for *C. jejuni*. Notably, we identified important differences regarding the presence of known and putative T3SS effector proteins in *C. jejuni* clinical strains isolated from Chile. One of the major differences was the absence of FspA homologs in most Chilean strains. FspA proteins are secreted by the fT3SS, and FspA1 induces apoptosis to host-cells *in vitro* (Poly et al., 2007). Therefore, the absence of these proteins may impact the virulence of these strains. Besides known fT3SS effector genes, we also detected important differences regarding the presence of 57 recently identified T3SS effector candidates (Gabbert et al., 2023). Based on the presence/absence of these putative effectors, the hierarchical analysis revealed five major subgroups with distinct effector repertoires (**Figure 5**). Different fT3SS effector repertories could translate to different infection outcomes due to differences in the ability of these strains to invade and colonize the gut. In addition, some of the most conserved effectors of the fT3SS (**Figure 5**) are poorly characterized highlighting the need to dissect the contribution of these effectors to the pathogenesis of *Campylobacter*.

We also observed significant differences regarding the presence of the T6SS and the distribution of its putative effector proteins. T6SS has emerged as a potential new virulence factor of *Campylobacter* spp (Liaw et al., 2019; Ungureanu et al., 2019), and was detected more frequently compared to our previous work (Bravo et al., 2021). Notably, the presence of T6SS was associated with strains of the chicken-specific CC-353. A lineage-relatedness was also reported by Rokney et al. with a high prevalence of T6SS in CC-353, CC-460, CC-607, and CC-446 (Rokney et al., 2018) and by Van Vliet in CC-464, CC- 353, CC-573 and CC-403 (van Vliet et al., 2021). Even though there is no evidence that this system is related to more severe clinical outcomes (Agnetti et al., 2019), its presence in *C. jejuni* strains from chicken-associated clonal complexes suggests that it might play a role in the increased ability to colonize the gastrointestinal tract of humans and chickens.

Regarding the presence and distribution of T6SS effector proteins, we observed significant differences between the Chilean clinical strains and type strain 488. The hierarchical analysis identified four distinct effector protein profiles, different from strain 488. Differences that may impact the ability of these clinical strains to compete with other bacteria during gut colonization in both chicken and humans. One group (IV) lacked most of the predicted effectors for this strain. We identified a putative new E/I module within the variable region of the T6SS gene cluster of strain CFSAN122739 (**Figure 6**). This module was present in most strains of subgroup I of the hierarchical analysis (**Figure 7**), suggesting that at least some clinical strains could harbor novel effectors beyond the repertoire predicted for the type 488 strain. The differences observed for both fT3SS and T6SS effector protein candidates could account for strain-dependent differences in pathogenicity and the ability of these strains to survive in the environment or colonize different hosts.

## 5 Final remarks

In conclusion, the study demonstrated the emergence of certain clones of *C. jejuni*, not previously identified in the Central region of Chile, and the presence of lineage-related virulence factors that might be relevant for transmission and/or clinical outcomes. More genomic studies are necessary to gain further information on the transmission dynamics of pathogenic *Campylobacter* spp. to humans and the potential emergence of new virulence and antimicrobial resistance markers in Chile and other Latin American countries.

## Data availability statement

The datasets presented in this study can be found in online repositories. The names of the repository/repositories and accession number(s) can be found in the article/**Supplementary Material**.

## Author contributions

AK: Methodology, Formal analysis, Investigation, Visualization, Resources, Data curation, Writing-Original Draft and review and editing. LP: Investigation, Visualization, review and editing final manuscript. TW: Investigation, Visualization, review and editing final manuscript. CV: Investigation, review and editing final manuscript. CM-R: Formal analysis, Investigation, Visualization, review and editing final manuscript. JU: Formal analysis, Investigation, Visualization, review and editing final manuscript. NG-E: Methodology, Formal analysis, Investigation, Visualization, Resources, Data curation, review and editing final manuscript. CG: Methodology, Formal analysis, Investigation, Visualization, Resources, Data curation, review and editing final manuscript. CB: Methodology, Formal analysis, Investigation, Visualization, Resources, Data curation, Funding acquisition, Writing-Original Draft and review and editing. VB: Conceptualization, Methodology, Formal analysis, Investigation, Visualization, Resources, Data curation, Writing-Original Draft and review and editing, Funding acquisition, Supervision and Project administration. All authors contributed to the article and approved the submitted version.

## Supplementary material

Supplementary Figure 1 Blast-based dendrograms of clinical *Campylobacter* strains harboring the pVir plasmid.

Supplementary Figure 2 Analysis and sequence alignment of the putative T6SS effector protein Z_00323 by the structure-based homology tool HHpred.

Supplementary Figure 3 Comparison of CCs distribution and antimicrobial resistance determinants in two periods of study in clinical *Campylobacter* strains isolated in Santiago, Chile. **(A)**. Comparison of clonal complexes distribution amongst clinical *Campylobacter* strains in two periods of study (2017-2019 vs. 2020-2021). Colored bars represent the different CCs described in both periods of study. **(B)**. Comparison of fluoroquinolones (azure), macrolides (pea) and tetracycline (yellow) resistance levels in the studied periods (2017-2019 vs. 2020-2021).
