# Supplementary figures and images for: Whole-genome sequencing reveals changes in genomic diversity and distinctive repertoires of T3SS and T6SS effector candidates in Chilean clinical Campylobacter strains
Source: Front Cell Infect Microbiol. 2023 Jul 13;13:1208825. doi: 10.3389/fcimb.2023.1208825 (PMC10374022; doi:10.3389/fcimb.2023.1208825)

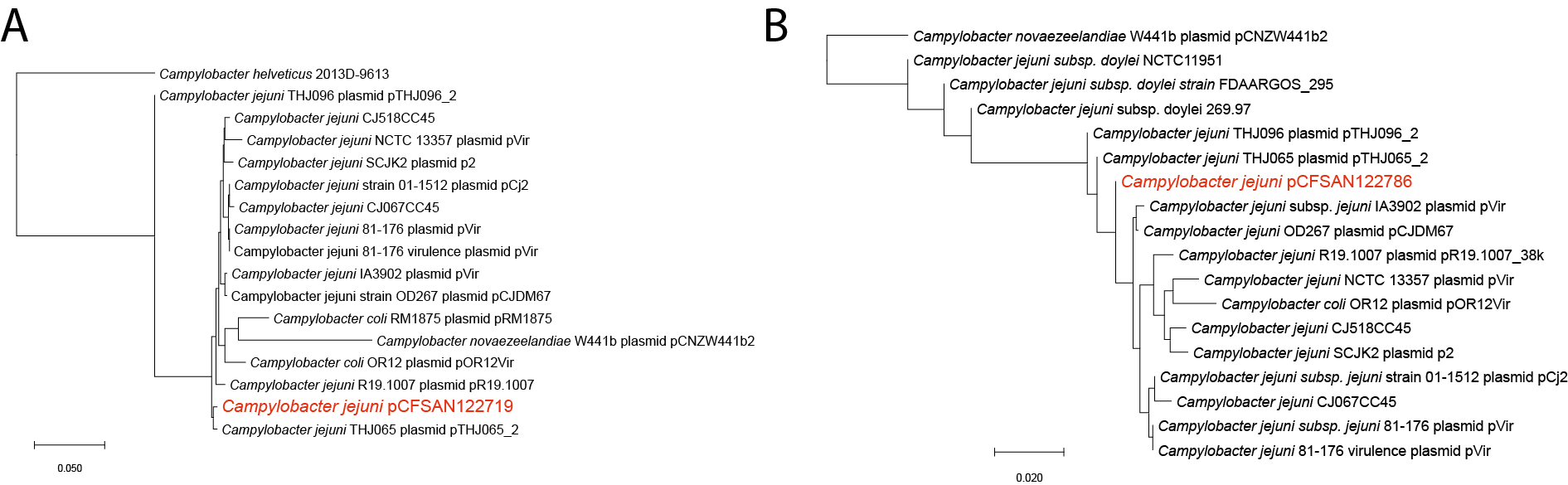

Supplement: Supplementary Figure 1 — Blast-based dendrograms of clinical Campylobacter strains harboring the pVir plasmid. [file Image_1.tif]

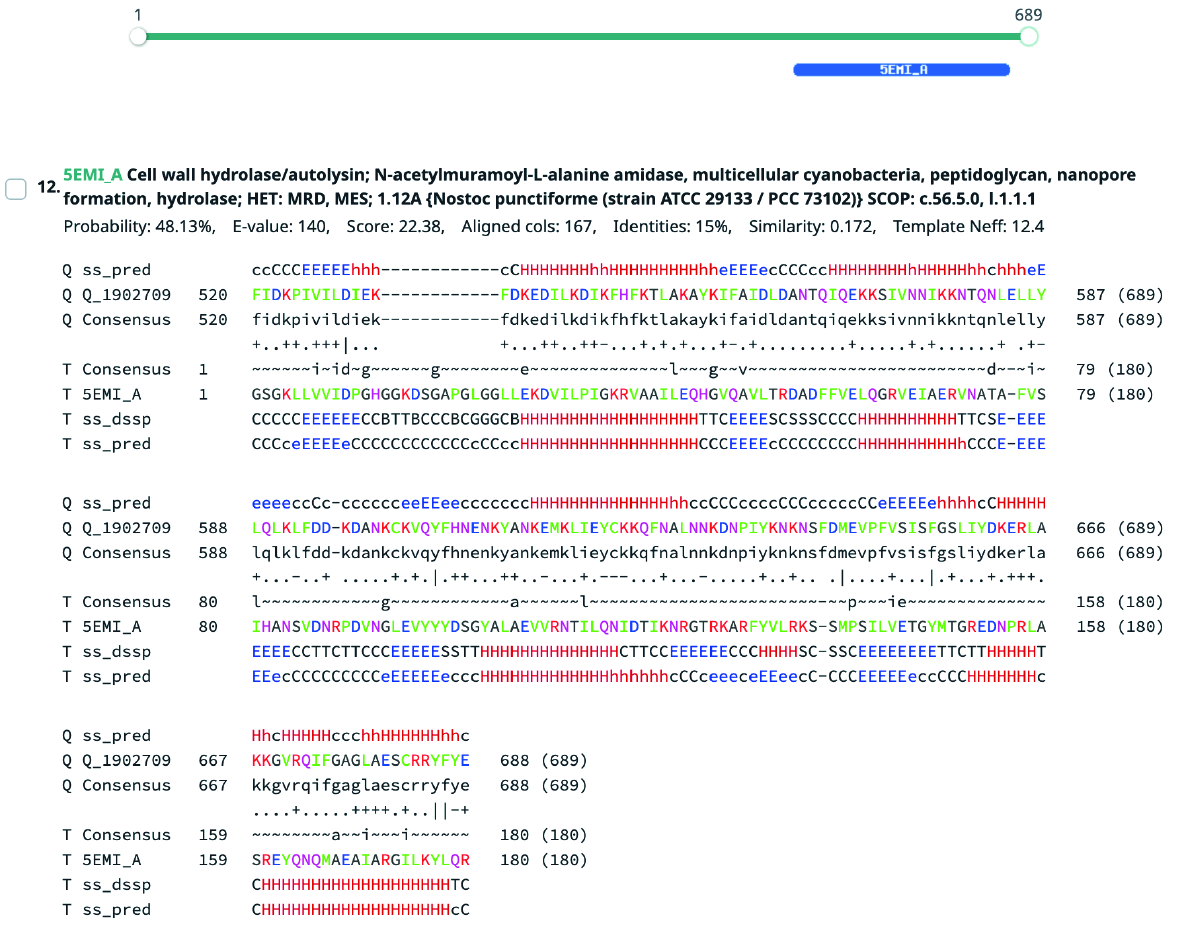

Supplement: Supplementary Figure 2 — Analysis and sequence alignment of the putative T6SS effector protein Z_00323 by the structure-based homology tool HHpred. [file Image_2.tif]

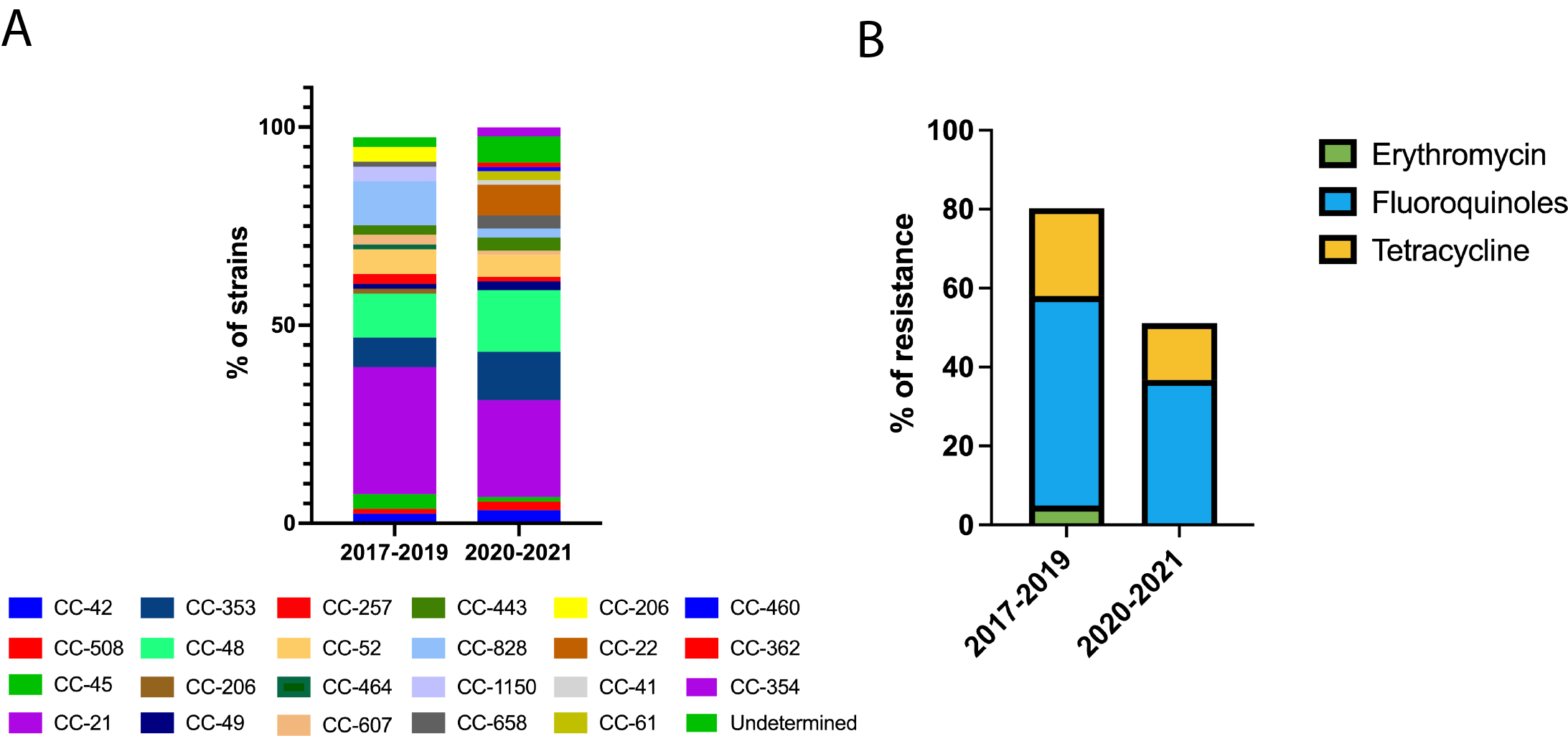

Supplement: Supplementary Figure 3 — Comparison of CCs distribution and antimicrobial resistance determinants in two periods of study in clinical Campylobacter strains isolated in Santiago, Chile. (A). Comparison of clonal complexes distribution amongst clinical Campylobacter strains in two periods of study (2017-2019 vs. 2020-2021). Colored bars represent the different CCs described in both periods of study. (B). Comparison of fluoroquinolones (azure), macrolides (pea) and tetracycline (yellow) resistance levels in the studied periods (2017-2019 vs. 2020-2021). [file Image_3.tif]
